# Case Report: Digital analysis of occlusion with T-Scan Novus in occlusal splint treatment for a patient with bruxism

**DOI:** 10.12688/f1000research.72951.1

**Published:** 2021-09-13

**Authors:** Dobromira Shopova, Tanya Bozhkova, Svetlana Yordanova, Miroslava Yordanova

**Affiliations:** 1 Department of Prosthetic Dentistry, Faculty of Dental Medicine, Medical University Plovdiv, Plovdiv, 4000, Bulgaria; 2 Department of Orthodontics, Faculty of Dental Medicine, Medical University, Plovdiv, 4000, Bulgaria

**Keywords:** T-Scan Novus, digital splint design, 3Shape, bruxism

## Abstract

Bruxism is a disease with a multifactorial etiology. Its clinical manifestations are most often an unaesthetic smile with abraded tooth surfaces, temporomandibular disorders and muscle hyperactivity. Here we present a case of bruxism where proper articulation of the occlusal splint was performed using the T-scan Novus system.

A patient with bruxism underwent treatment with stabilization splint made by 3D printer technology. Intraoral scanning was performed using Trios Color (3Shape, 2014), and the digital design was achieved using the 3Shape Dental system design - splint studio. Formlabs Form 2 printer with biocompatible resin Dental LT Clear Resin was used for printing. The T-Scan Novus system with software attached to it, version 9.1, was used for digital examination of the occlusion. A 2.7 mm thick occlusal splint was developed, and the software adapted the occlusion with antagonists. After adjustment with T-Scan Novus, a reduction in disocclusion time of the patient was achieved, which is a desired result in the treatment of bruxism. The position of the joint components was proven radiologically.

The treatment of bruxism with splint therapy continues to be the main method of treatment. Using digital technology allows for more accurate constructions and precise balancing of occlusal relationships.

## Introduction

Bruxism is a disease with a multifactorial etiology, such as stress, occlusal factors and trauma. Its clinical symptoms are most often abraded tooth surfaces, muscle hyperactivity, temporomandibular joint (TMJ) disorders (pain, clicking, limited opening), and in advanced cases headache and hearing problems can be observed. ^1^

The treatment of bruxism is reduced to several main methods - increasing the vertical dimension of occlusion (VDO) to normal, medialization of the mandible to influence the position of the joint condyle in the joint fossa and release the disc, and positioning the mandible in a balanced stable occlusion. ^2, 3^ This is achieved by splint therapy for a certain adaptation period and then the result can be fixed by orthodontic treatment, adhesive restorations or prosthetic construction. ^4^ The stabilization splint is indicated for the most common symptoms of TMJ and muscle. ^2^

Digital technologies for dental purposes have undergone enormous development in recent years. Brand giants have developed laboratory protocols from the initial unit of digital model creation (via intraoral or laboratory scanner), through to the modeling of substructures (via design software) to the actual creation of the final product (using 3D printing and CAD/CAM methods). Modern dentistry is strongly influenced by digital technologies. ^5- 7^ Innovative technologies already have an established protocol for creating splints with personal design, from different materials and by different methods. ^8- 10^

Achieving a balanced occlusion requires a number of evenly distributed contacts. ^11^ Articulation paper is widely used in practice when registering occlusal contacts. The interpretation of the markers obtained with articulation paper hides the possibility of making mistakes, as it is subjective. It is wrong to perceive large and dark markers as the places with the highest load. ^12^ With articulation paper, it is only possible to locate the contacts. The T-Scan Novus digital occlusion analysis system is used to study the sequence of occurrence and the strength of the occlusal contacts. Created 35 years ago by the Maness, this system has proven its usefulness in the study of occlusal contacts. When using T-Scan, obtained information allows accurate occlusal adjustment. The registered contacts are displayed as 2D and 3D images, and are colored in different colors depending on the applied force. The system automatically calculates occlusion time (OT) and disocussion time (DT). The OT is the elapsed time in seconds, measured from the first tooth contact until the last tooth contact. Maximum intercuspation always occurs before the patient achieves maximum bite force. The OT describes the degree of bilateral time simultaneity present in a patient occlusion. The ideal duration of OT is ≤0.2 s. The DT is the elapsed time in seconds, measured from the beginning of an excursive movement made in any direction (left, right, or forward) with all teeth in maximum intercuspation until only canines and/or incisors are in contact. The ideal duration of DT is ≤0.5 s. ^13^ Kerstein and Wright ^14^ were the first to suggest that some patients with temporomandibular disorders, including those with bruxism, can be treated by reducing DT. They correct DT using a technique they call immediate complete anterior guidance development (ICAGD). This approach to occlusal therapy establishes immediate posterior disocclusion in all mandibular movements, prior to any habitual closure adjustments. ^15^ Patients with myofascial pain have been shown to have increased DT. By the decreasing of DT, it reduces the hyperactivity of masseter and temporalis, the main muscles closing the lower jaw. ^15, 16^

Here, we report a patient with bruxism, where proper articulation of the occlusal splint was performed using the T-scan Novus System.

## Case report

### Initial presentation

**Figure 1.**
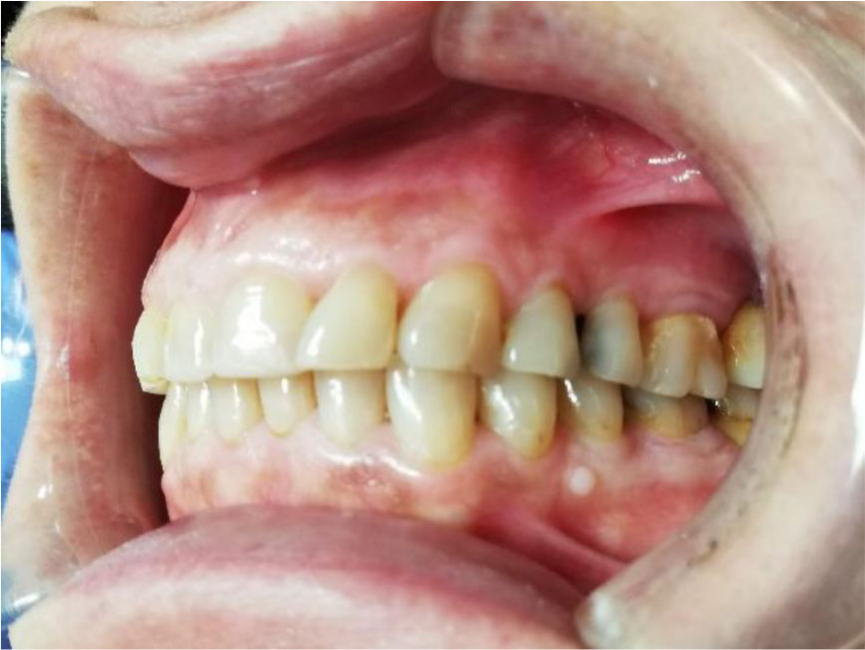
Initial presentation showing central occlusion.

**Figure 2.**
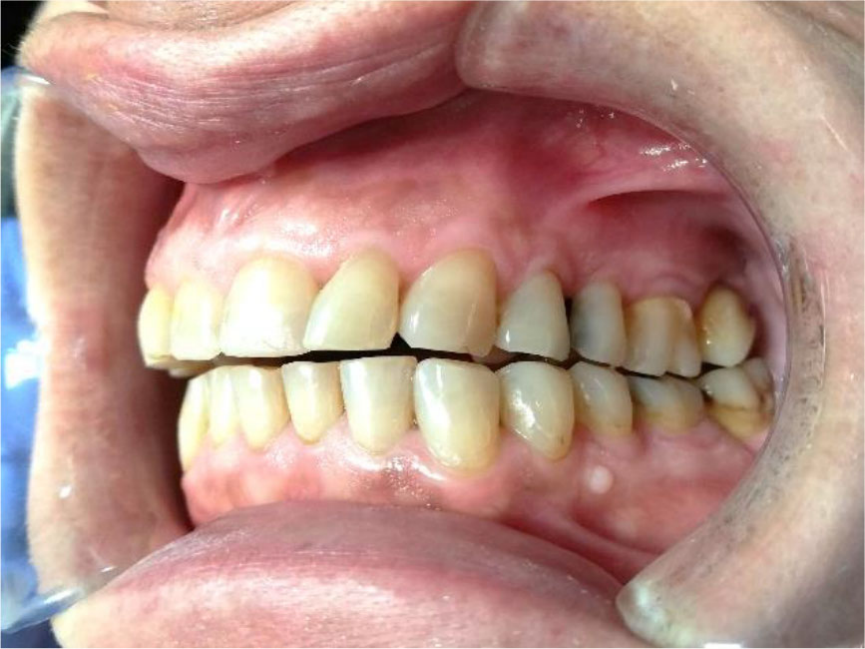
Initial presentation showing abraded teeth surfaces.

A 56-year-old female patient, Bulgarian shop assistant, underwent treatment for bruxism. The leading complaints were an unaesthetic appearance of the smile, muscle fatigue almost all day and the clicking of both TMJ. Intraoral examination revealed highly abraded tooth surfaces entering the dentin area and a flattened smile line ( Figures 1 and 2). The main masticatory muscles, masseter and temporalis, had increased tone, and the patient reported headaches related to this. On palpation of the TMJ, the patient responded with mild to moderate pain. There was a sharp click as the jaw was opened wide.

We decided to treat the patient with 3D printed stabilization splints. For this purpose, a digital work protocol was applied. An intraoral scan was performed with a Trios Color scanner (3Shape). The splint protocol requires imaging of the upper, lower jaw, left and right bite. The 3Shape Dental system design - splint studio was used for digital design. The splint was made using 3D printing with a Formlabs Form 2 printer and biocompatible resin (Dental LT Clear Resin).

The T-Scan Novus system with software attached to it, version 9.1, was used for digital examination of the occlusion. For the intraoral localization of the contacts, a two-stage technique was applied using Bausch 40 microns articulation paper and Bausch 12 microns articulation foil.

### Initial digital design of the splint

**Figure 3.**
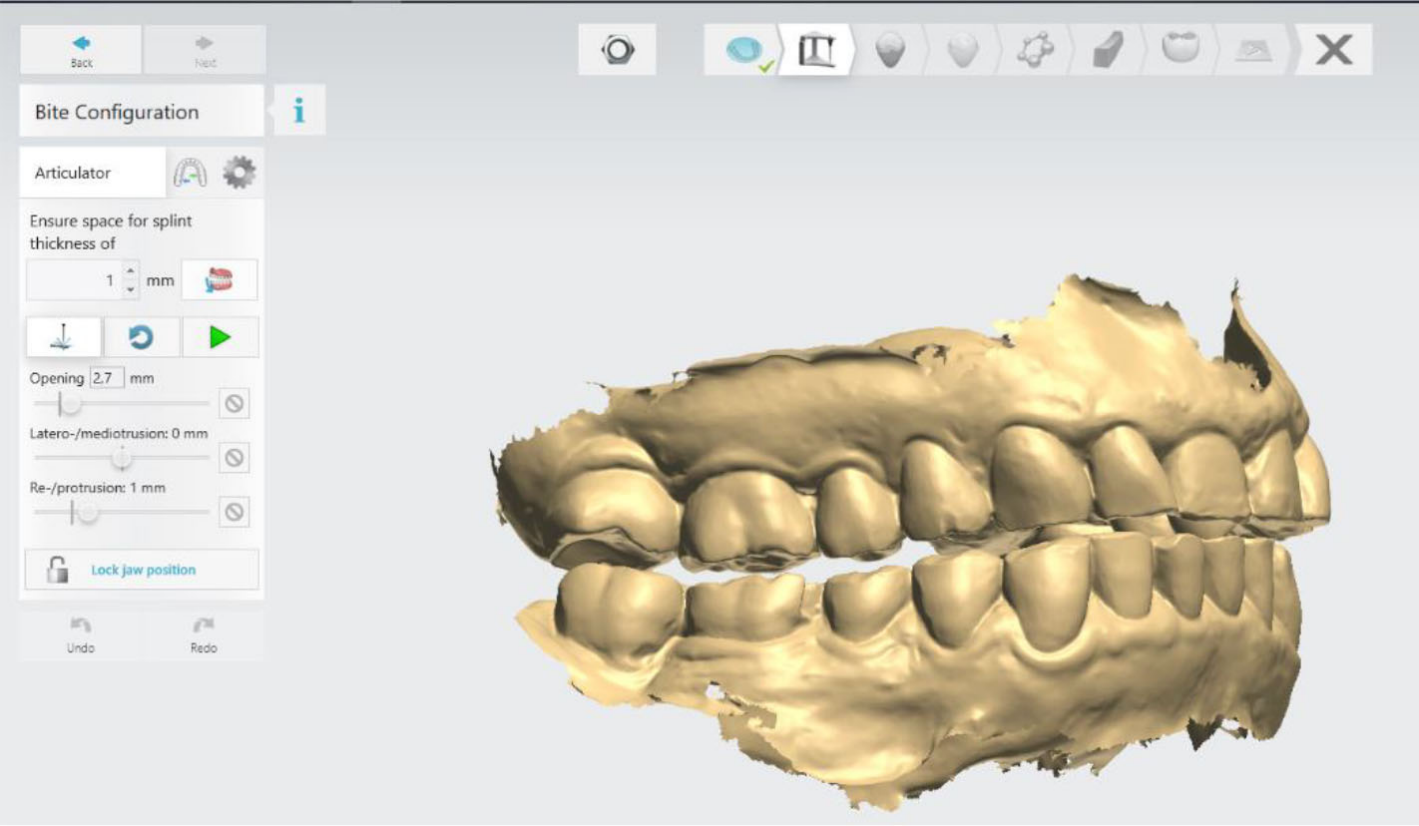
Intermaxillary relation for splint creation (3Shape).

The digital software allows different design options. In this case, the following parameters of the lower jaw position were set: vestibular thickness of the splint at 1 mm; occlusal opening at 2.7 mm; and protrusion at 1 mm ( Figure 3).

**Figure 4.**
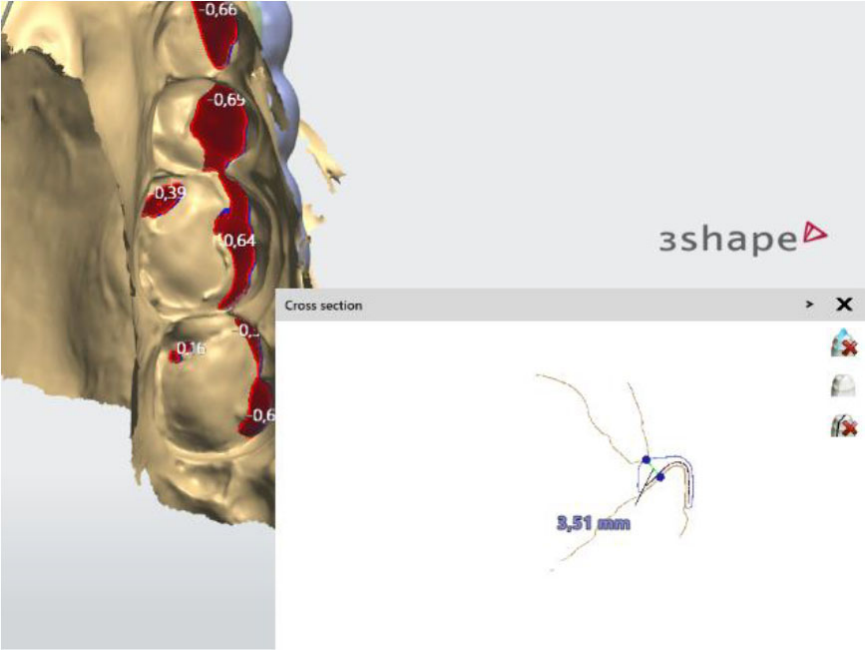
Frontal palatal extension of the splint (3Shape).

**Figure 5.**
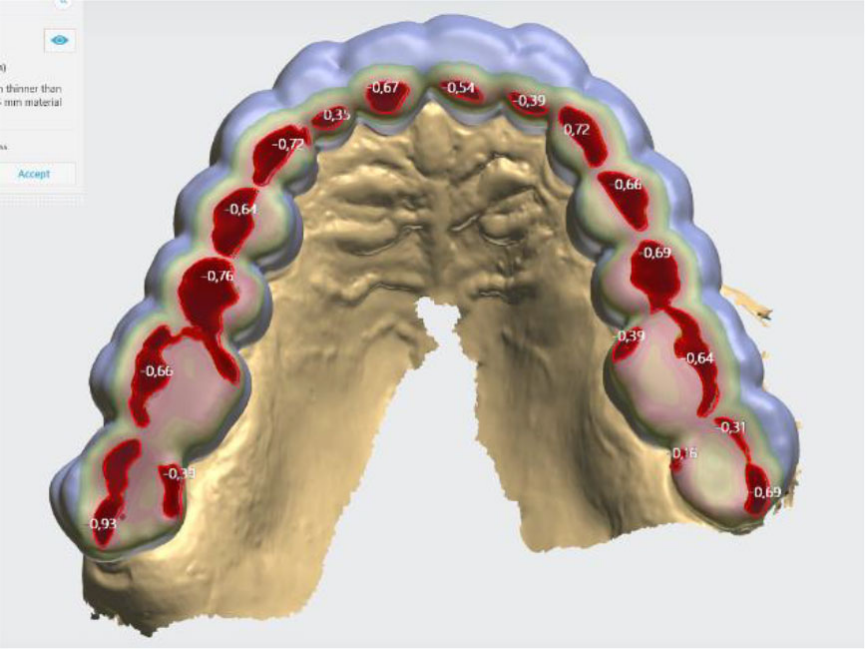
Balanced occlusion by the software 3Shape Splint studio.

When designing the occlusal surfaces, an option with relief to the antagonists was set. A surface modeled in this way has a slight occlusal relief, which leads the opposite jaw to the designed position. Occlusal surfaces were software-extended and provide wider contact. This is especially evident in the frontal teeth area. In this way a balanced stable occlusion was achieved. As the patient's clinical crowns were without retention areas, the splint borders were located to the cervical zone vestibularly and palatally, except the distal teeth, where the borders were 1 mm up of the cervix vestibularly. This extension also contributed to its stable position ( Figures 4 and 5).

**Figure 6.**
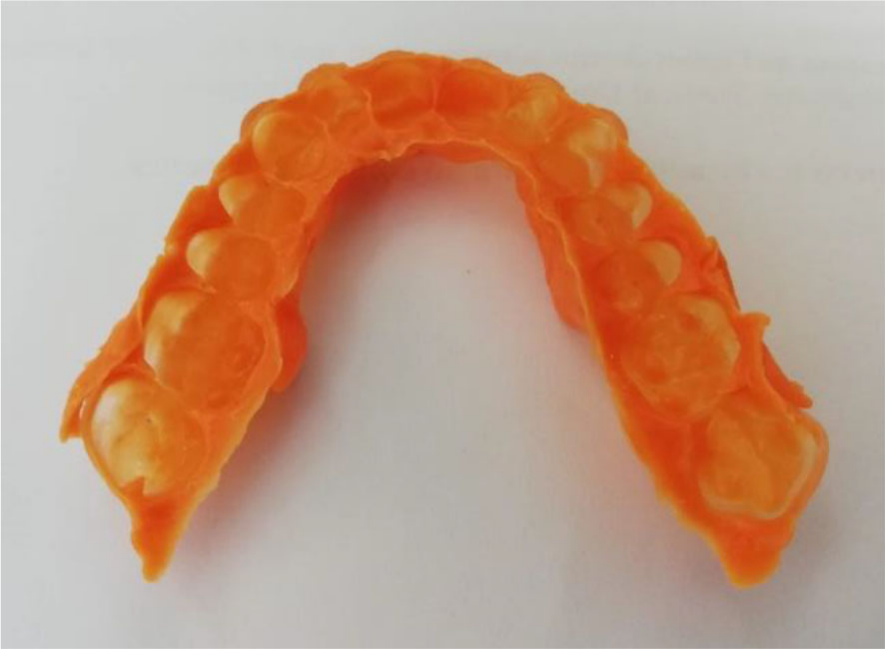
Silicon test for splint adaptation.

**Figure 7.**
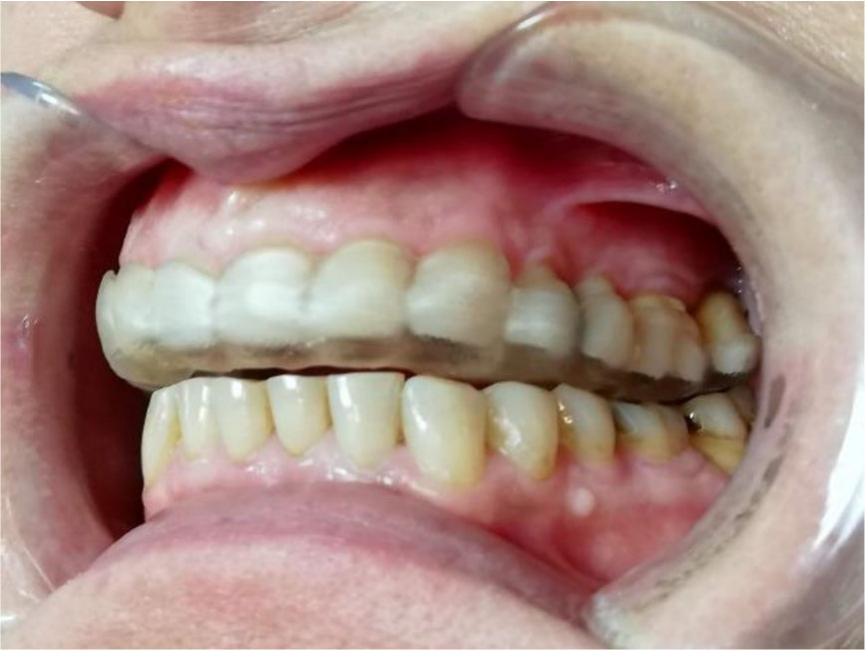
Stabilization splint in the patient’s mouth.

Once the splint had been designed, its adaptation to the tooth surfaces was checked by a silicone test ( Figures 6 and 7).

### Computer-guided occlusal splint adjustment

After proving its stable position on the tooth surfaces, we proceeded to computer-guided occlusal splint adjustment with the T-Scan Novus System.

**Figure 8.**
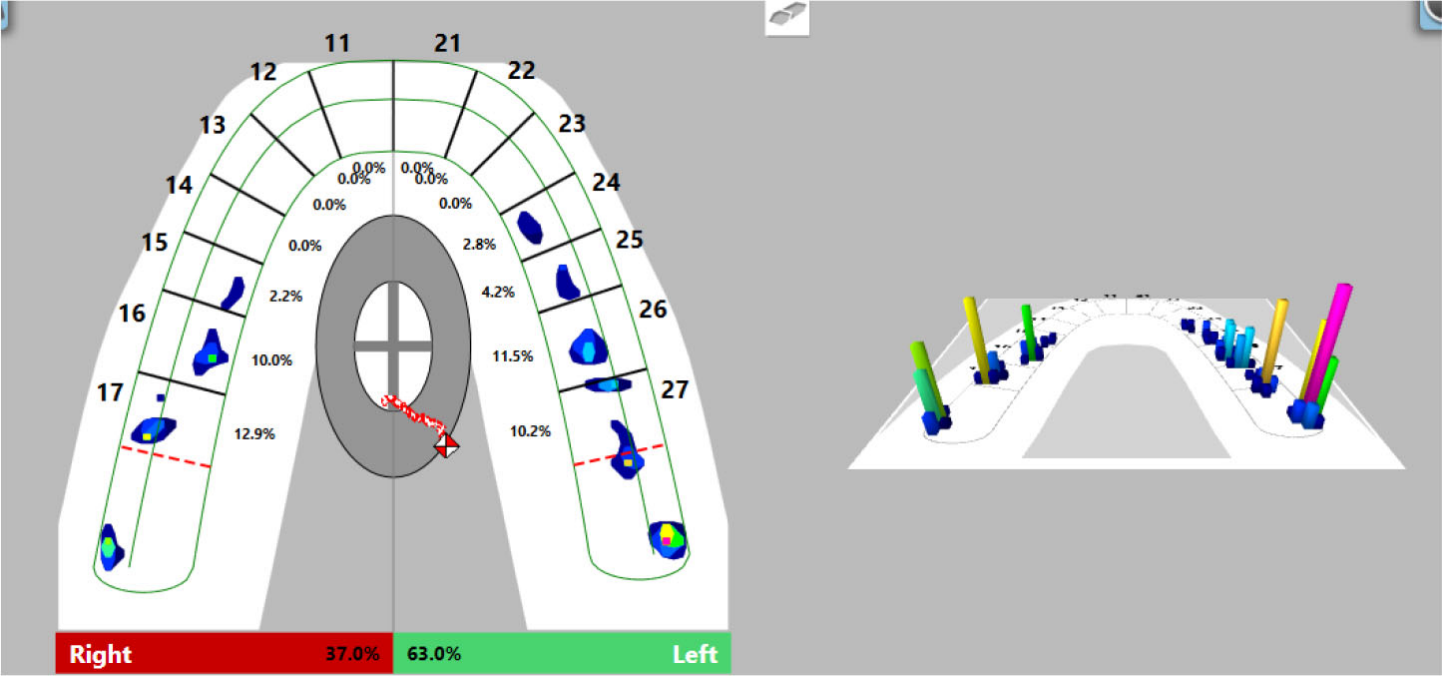
First occlusal registration (T-Scan Novus).

Firstly, we registered occlusal contacts with the splint in the mouth using the T-Scan Novus software. Registered occlusal contacts are represented as two-dimensional contour images or three-dimensional images. The strength of the contacts is determined in color by the help of a scale; weak contacts are colored in blue and strong contacts in red ( Figure 8). In our patient, uneven distribution of occlusal contacts with predominance of forces in the left side was established. In addition, center of force (COF) was shifted to the left. Elevated values of OT (0.52 s) and DT (0.74 s) were reported.

**Figure 9.**
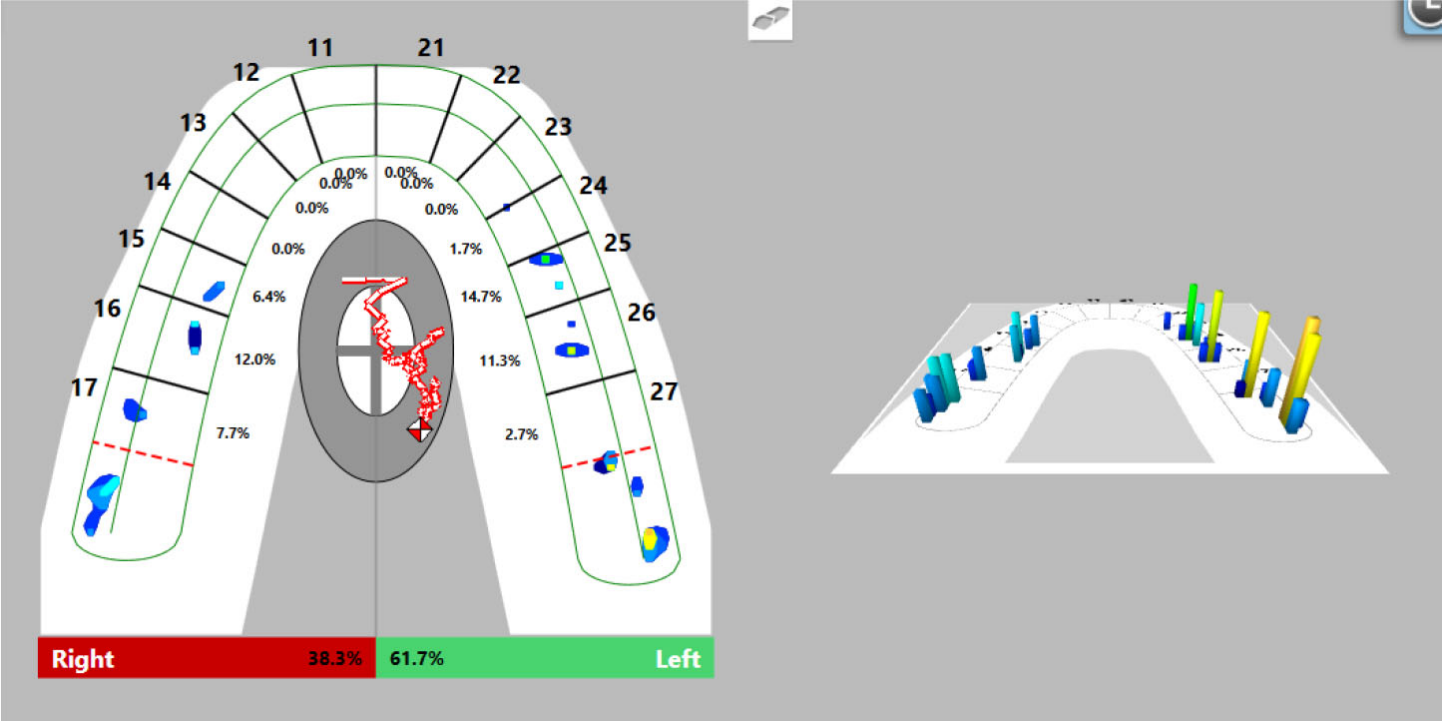
Intermediate occlusal registration (T-Scan Novus).

Visualization of the occlusal contacts was done intraorally with articulation paper and foil. After removing the strong contacts, a new record was made ( Figure 9). This revealed that a greater load was still received by the left side, but the forces were reduced. The COF was still shifted to the left, at the periphery. Reduced OT values (0.47 s) were detected and DT values (0.4 s) were normalized.

**Figure 10.**
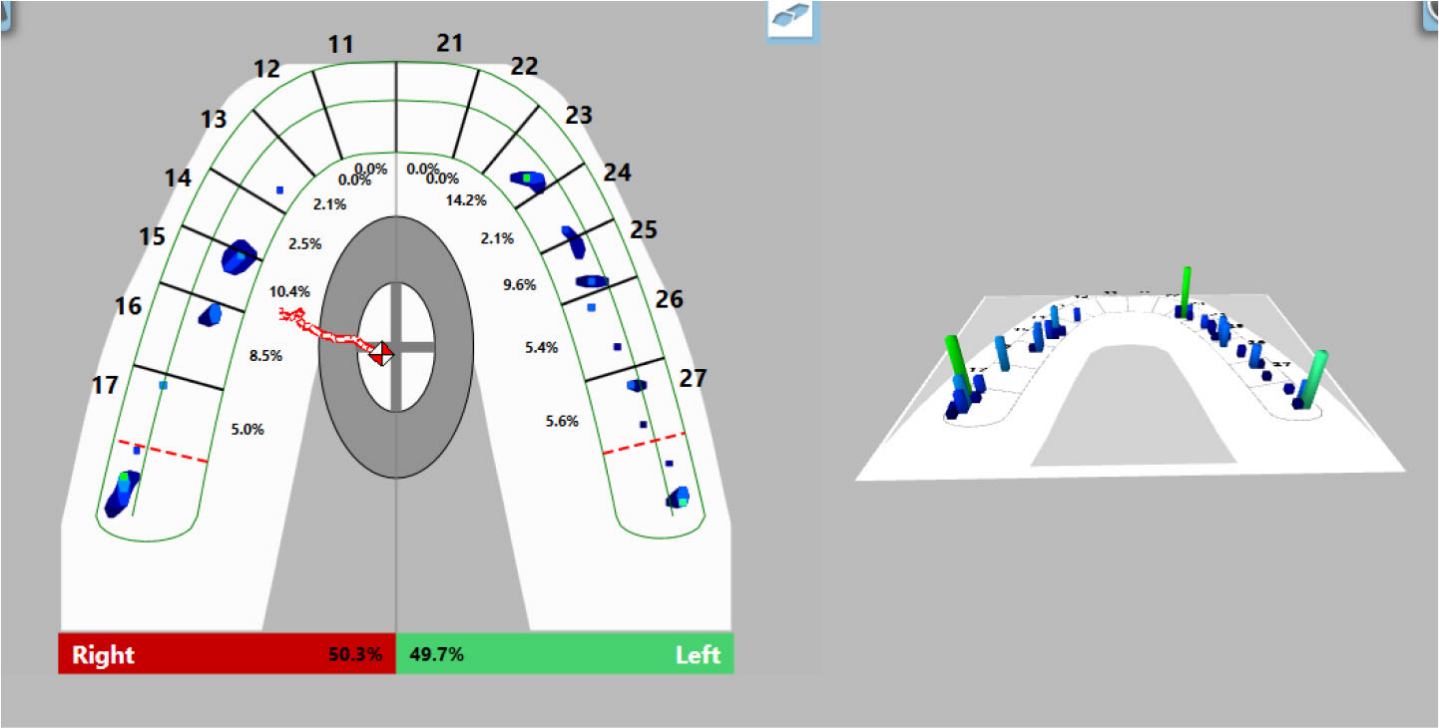
Final occlusal registration (T-Scan Novus).

On the last recording of the occlusion, evenly distributed contacts in the area of the lateral teeth were established. The forces on both sides are approximately equal (right side 50.3%, left side 49.7%) and COF was in the middle. The values of OT and DT were normal (0.27 s and 0.4 s, respectively) ( Figure 10).

**Figure 11.**
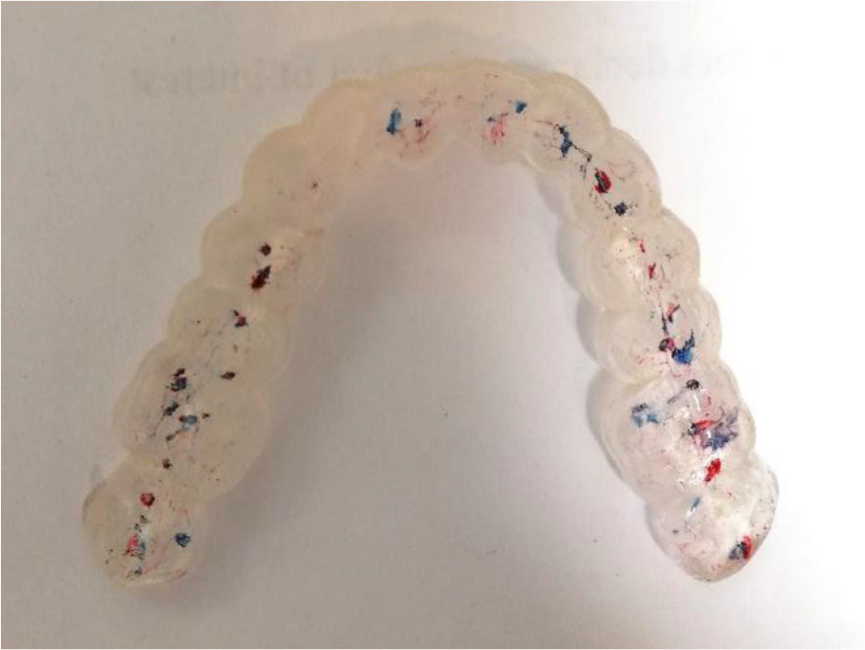
Occlusal registration with thin foil and articulation paper.

The occlusion of the patient visualized by a two-stage technique (Bausch 40 microns articulation paper and Bausch 12 microns articulation foil) almost confirmed the result of the digitally obtained result. The frontal area was marked with contacts with the articulation paper and foil, which weren’t registered by the T-Scan Novus System ( Figure 11).

### Follow-up

**Figure 12.**
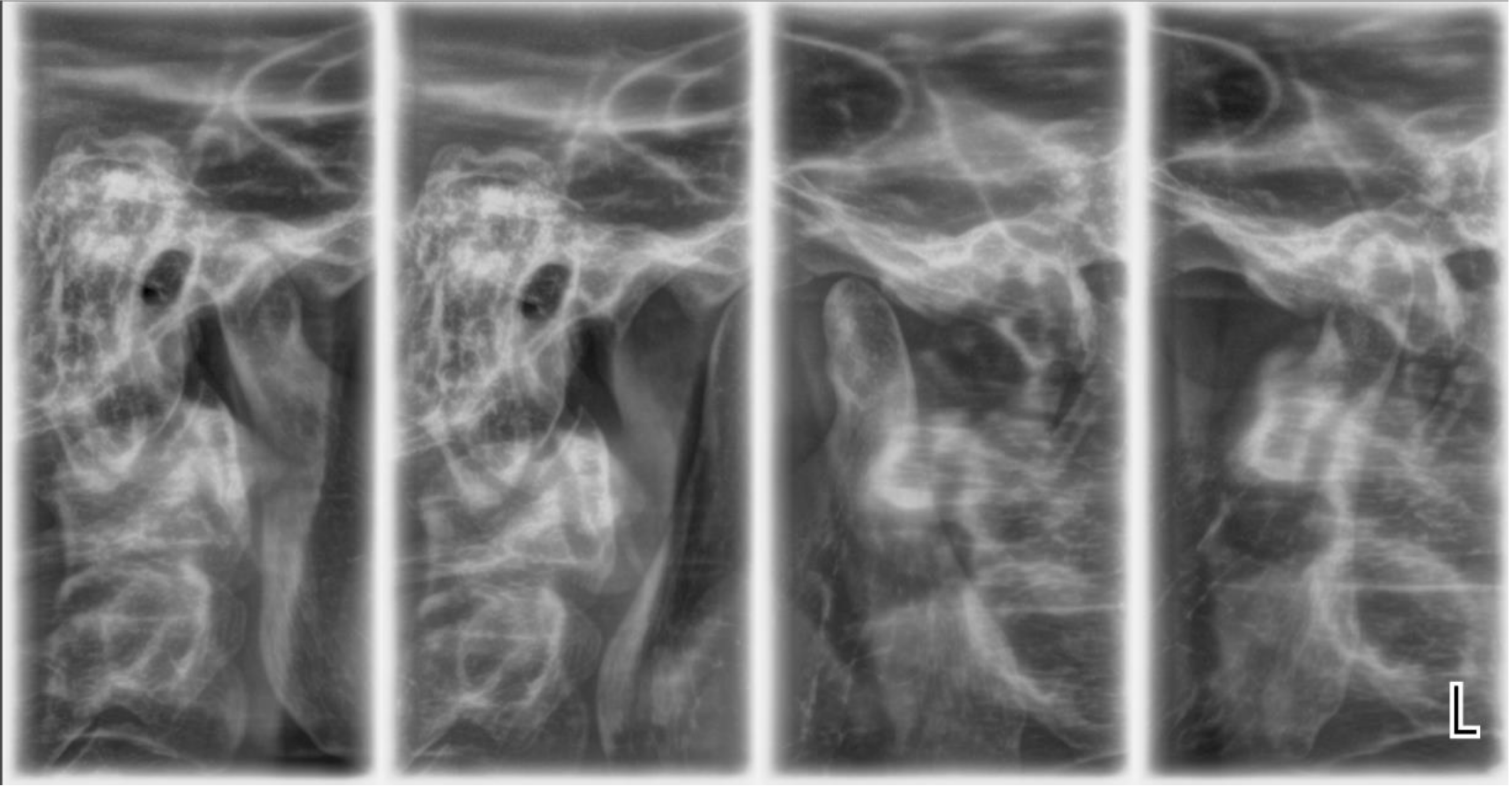
TMJ in open and closed position.

**Figure 13.**
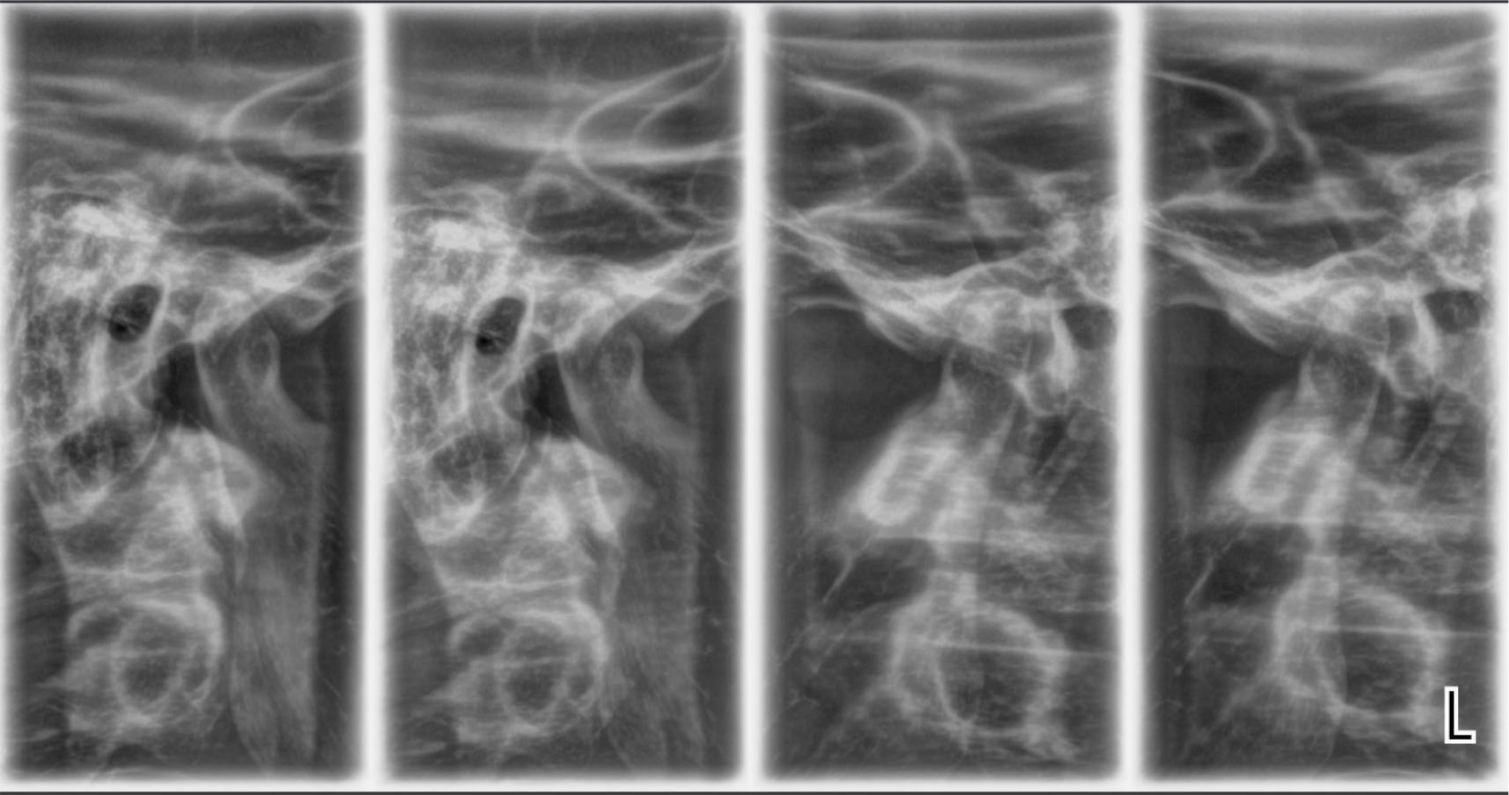
TMJ condition with the splint, and with the splint and spacer 1.5 mm.

A radiological examination was performed to check the condition of the TMJ. Four positions of the joint were made: in open and closed position, with the splint, and with the splint and a 1.5 mm spacer ( Figures 12 and 13).

The radiological exam shows that there was symmetry between the right and left TMJ, no structural changes of the articular condyle and eminence were detected, the joint condyle was located symmetrically in the joint capsule, and when the mouth was opened, the condyle passes the eminence, the so-called habitual luxation.

## Discussion

The treatment of bruxism is difficult and long. Visual evidence is needed that a treatment plan is working. T-Scan Novus visualizes the contacts between the jaws, the movement of the lower jaw, as well as establishes values of parameters (OT and DT), which are of major importance for the treatment of bruxism.

Stabilization splints for patients with bruxism aim to raise TMJ pain, muscle hyperactivity and restore VDO. ^2, 3^ Extending occlusal surfaces contributes to a more stable positioning of the lower jaw. The lack of blocks and preliminary contacts favors the final result of the treatment. ^2, 4^

Digital design enables a very good and even adaptation of the splint to the tooth surfaces to be achieved. Occlusal surface was less precise. The reason can be found in the minimum output thickness of the material to be made (for 3D printing), which the software automatically sets. When using a splint milling by CAD/CAM technology, the mismatch can be sought again in the minimum thickness of the material, but also the limitation of the thinnest bur for cutting. ^6- 8^

The T-Scan Novus System shows more than just occlusal contacts. In the case of bruxism, it is more valuable to rely on OT and DT. In our study, during the clinical adjustment, a reduction of the values was achieved by almost half; the reported OT and DT approached the norm (OT: 0.27 s, norm ≤0.2 s; DT: 0.4 s, norm ≤0.5 s). The achieved result (DT) is a proven method of treatment for bruxism. ^14, 15^

In our study, visualization of the registered intraoral occlusal contacts was performed by a two-stage technique with articulation paper 40 microns and articulation foil 12 microns. When comparing the obtained results with those of the T-Scan Novus system, differences in the number and location of the occlusal contacts were established. This proves the possibility of registering false positive contacts when using articulation paper. ^12^

Radiological examination is the only option for objective examination of TMJ. As an initial study it serves to analyze the anatomical structures, their location relative to each other, and to exclude other causes of joint pain (trauma, ankylosis, tumor disease). X-rays taken after splinting prove the position of the joint components. In case of unsatisfactory or irrelevant placement, the splint should be redesigned. ^1, 4^

## Conclusion

The main method of bruxism treatment continues to be splint therapy. Its combination with digital technologies allows more precise constructions and more detailed visualizations at each stage - design and dental adaptation of the splint, occlusal relief and occlusal relationships. As shown in our patient, with the T-Scan Novus system, it is possible to achieve optimal, harmonious occlusal ratios.

## Consent

Written informed consent was obtained from the patient for the publication of the case report with any associated images.

## Data availability

All data underlying the results are available as part of the article and no additional source data are required.

## References

[1] Okeson JP : Management of Temporomandibular Disorders and Occlusion-E-Book. *Elsevier Health Sciences.* 2019.

[2] Al-Ani M Ziad : Stabilisation splint therapy for temporomandibular pain dysfunction syndrome. *Cochrane Database of Systematic Reviews* .2004;1. 10.1002/14651858.CD002778.pub2 14973990

[3] Lakshmi MS : Occlusal Splint Therapy in Temporomandibular Joint Disorders: An Update Review. *J International Oral Health* .2016;8(5). 10.1002/14651858.CD012850

[4] Bumann A Lotzmann U : *TMJ Disorders and Orofacial Pain* . Stuttgart, New York: Thieme;2002.

[5] Dawood A Marti BM Sauret-Jackson V : 3D printing in dentistry. *Br Dental J.* 2015;219(11):521–529. 10.1038/sj.bdj.2015.914 26657435

[6] Mikolajczyk T Malinowski T Moldovan L : CAD CAM system for manufacturing innovative hybrid design using 3D printing. *Procedia Manufacturing.* 2019;32:22–28. 10.1016/j.promfg.2019.02.178

[7] Zhou Q Wang Z Chen J : Development and evaluation of a digital dental modeling method based on grating projection and reverse engineering software. *J Prosthetic Dentistry.* 2016;115(1):42–46. 10.1016/j.prosdent.2015.06.016 26384536

[8] Edelhoff D : CAD/CAM splints for the functional and esthetic evaluation of newly defined occlusal dimensions. *Quintessence Int.* 2017;48.3. 10.3290/j.qi.a37641 28232961

[9] Nota A Ryakhovsky AN Bosco F : A Full Digital Workflow to Design and Mill a Splint for a Patient with Temporomandibular Joint Disorder. *Appl. Sci.* 2021;11(1):372. 10.3390/app11010372

[10] Venezia P Muzio LL De Furia C : Digital manufacturing of occlusal splint: From intraoral scanning to 3D printing. *J Osseointegration.* 2019;11(4):535–539. 10.23805/JO.2019.11.03.10

[11] Atashrazm P Lari HA Khorsand M : An Evaluation of Occlusal Contacts of Remounted Complete Denture before Final Occlusal Adjustment. *J Dentistry. Shiraz University of Medical Sciences.* 2009;9:1–5.

[12] Carey J Craig M Kerstein RB : Determining a relationship between applied occlusal load and articulation paper mark area. *Open Dent J.* 2007;1:1–7. 10.2174/1874210600701010001 19088874 PMC2581523

[13] Kerstein R : Handbook of research on computerized occlusal analysis technology applications in dental medicine. *IGI global.* 2014:1–15.

[14] Kerstein RB Wright NR : Electromyographic and computer analyses of patients suffering from chronic myofascial paindysfunction syndrome: before and after treatment with immediate complete anterior guidance development. *J Prosthet Dent.* 1991;66:677–686. 10.1016/0022-3913(91)90453-4 1805009

[15] Kerstein RB : Disocclusion time-reduction therapy with immediate complete anterior guidance development to treat chronic myofascial pain-dysfunction syndrome. *Quintessence Int.* 1992;23:735–747. 1305288

[16] Kerstein RB Lowe M Harty M : A force reproduction analysis of two recording sensors of a computerized occlusal analysis system. *Cranio.* 2006;24:15–24. 10.1179/crn.2006.004 16541841

[17] Kerstein RB : Reducing chronic masseter and temporalis muscular hyperactivity with computer-guided occlusal adjustments. *Compend Contin Educ Dent.* 2010;31:530–534. 536538. 20879206

