# Supplementary material for: Case Report: Digital analysis of occlusion with T-Scan Novus in occlusal splint treatment for a patient with bruxism
Source: F1000Res. 2022 Jan 13;10:915. Originally published 2021 Sep 13. [Version 2] doi: 10.12688/f1000research.72951.2 (PMC8758972; doi:10.12688/f1000research.72951.2)
Supplement: Peer review history

# Peer review: Reviewer response for version 2

**DOI:** 10.5256/f1000research.119992.r119614

**Published:** 2022-01-13

**Authors:** Dinesh Rokaya

**Affiliations:** 1 Department of Clinical Dentistry, Walailak University International College of Dentistry, Walailak University, Bangkok, Thailand

The article is improved and I have no further comments.

Are enough details provided of any physical examination and diagnostic tests, treatment given and outcomes?

Yes

Is the case presented with sufficient detail to be useful for other practitioners?

Partly

Is sufficient discussion included of the importance of the findings and their relevance to future understanding of disease processes, diagnosis or treatment?

Partly

Is the background of the case’s history and progression described in sufficient detail?

Yes

Reviewer Expertise:

Clinical dentistry, prosthetic dentistry, digital dentistry, dental biomaterials

I confirm that I have read this submission and believe that I have an appropriate level of expertise to confirm that it is of an acceptable scientific standard.

# Peer review: Reviewer response for version 1

**DOI:** 10.5256/f1000research.76564.r99206

**Published:** 2021-11-18

**Authors:** Dinesh Rokaya

**Affiliations:** 1 Department of Clinical Dentistry, Walailak University International College of Dentistry, Walailak University, Bangkok, Thailand

This article presents the management of bruxism with occlusal splint with adjustment done using the T-scan Novus system.

**Abstract:**

- Please mention which Trios was used, 3 or 4.

**Introduction:**

- At present, although various scanners are available in the market, the Trios system presents the best scan which system is used in this case study.
- The authors need to add the following current literature in the introduction:
  - Amornvit *et al*., 2021 ^1^ - This study compared the various intraoral scanners and the results show that Trios showed the best results.
  - Amornvit *et al*., 2020 ^2^ - This study explained the confocal technique used in Trios which showed high accuracy especially in the posterior region.

**Case Report:**

- It is better to add details of the product and software such as Trios, resin, etc. Product name, Company, City, and Country.
- Figures 12 and 13: Each X-ray needs to be labeled.
- How often the recall was done and how long the splint was given needs to be mentioned.

**Discussion:**

- At present, scanning the upper arch, lower arch and bite allows to check the occlusion and force on the area of each tooth using 3Shape Dental System. This is also shown in this case. The occlusion can also be done more precisely using T-Scan but the disadvantage is this will add extra cost to the dentist and the patient. Hence, the authors need to add the specific use of T-Scan in this case study.
- In addition, the authors can discuss the indications and limitations of each system in checking the occlusion. This comparison can be done using a table. The following articles can be helpful: Lee *et al*., 2018 ^3^; Cheng *et al*., 2018 ^4^; Solaberrieta *et al*., 2016 ^5^.

Are enough details provided of any physical examination and diagnostic tests, treatment given and outcomes?

Yes

Is the case presented with sufficient detail to be useful for other practitioners?

Partly

Is sufficient discussion included of the importance of the findings and their relevance to future understanding of disease processes, diagnosis or treatment?

Partly

Is the background of the case’s history and progression described in sufficient detail?

Yes

Reviewer Expertise:

Clinical dentistry, prosthetic dentistry, digital dentistry, dental biomaterials

I confirm that I have read this submission and believe that I have an appropriate level of expertise to confirm that it is of an acceptable scientific standard, however I have significant reservations, as outlined above.

## References

[1] : Comparison of Accuracy of Current Ten Intraoral Scanners. *Biomed Res Int* .2021;2021: 10.1155/2021/2673040 2673040 10.1155/2021/2673040 34552983PMC8452395

[2] : Confocal 3D Optical Intraoral Scanners and Comparison of Image Capturing Accuracy. *Computers, Materials & Continua* .2020;66(1) : 10.32604/cmc.2020.011943 303-314 10.32604/cmc.2020.011943

[3] : Comparison of the occlusal contact area of virtual models and actual models: a comparative in vitro study on Class I and Class II malocclusion models. *BMC Oral Health* .2018;18(1) : 10.1186/s12903-018-0566-7 10.1186/s12903-018-0566-7 29921259PMC6009969

[4] : [Influence of intraoral scan and dental cast scan on occlusal quantitative analysis of virtual dental model]. *Beijing Da Xue Xue Bao Yi Xue Ban* .2018;50(1) :136-140 29483736

[5] : Intraoral Digital Impressions for Virtual Occlusal Records: Section Quantity and Dimensions. *Biomed Res Int* .2016;2016: 10.1155/2016/7173824 7173824 10.1155/2016/7173824 26881226PMC4736956

# Response

**Published:** 2021-12-02

**Authors:** dobromira shopova

Dear Reviewer, 

 Thank you for your advices! I will answer point by point:

**Abstract:**

- Please mention which Trios was used, 3 or 4.

**Author response:** The previous generation intraoral scanner of 3Shape Trios color, from 2014, was used.

**Introduction:**

- At present, although various scanners are available in the market, the Trios system presents the best scan which system is used in this case study.
- The authors need to add the following current literature in the introduction:
  - Amornvit *et al*., 2021 ^1^ - This study compared the various intraoral scanners and the results show that Trios showed the best results.
  - Amornvit *et al*., 2020 ^2^ - This study explained the confocal technique used in Trios which showed high accuracy especially in the posterior region.

**Author response: **I added them, thank you!

**Case Report:**

- It is better to add details of the product and software such as Trios, resin, etc. Product name, Company, City, and Country.

**Author response: **The information is added!

- Figures 12 and 13: Each X-ray needs to be labeled.

**Author response: **I labeled them, thank you!

- How often the recall was done and how long the splint was given needs to be mentioned.

**Author response: **After 3 months the recall was made. Clinical symptoms were reduced. The control study with T-Scan showed small changes, but within the same limits of OT and DT as the placement.

**Discussion:**

- At present, scanning the upper arch, lower arch and bite allows to check the occlusion and force on the area of each tooth using 3Shape Dental System. This is also shown in this case. The occlusion can also be done more precisely using T-Scan but the disadvantage is this will add extra cost to the dentist and the patient. Hence, the authors need to add the specific use of T-Scan in this case study.

**Author response: **Our study with the T-Scan system revealed an unbalanced distribution of occlusal contacts, although they were designed to be balanced by 3Shape Splint studio software. The reason can be found in the minimum output thickness of the material to be made (for 3D printing), and which the software automatically sets.

 There is evidence in the literature that one of the limitations of intraoral scanners is that they cannot measure the intensity of occlusal forces. The T-Scan system has been shown to be a reliable method for assessing the occlusal contact zone (Ayuso‐Montero, R., Mariano‐Hernandez, Y., Khoury‐Ribas, L., Rovira‐Lastra, B., Willaert, E., & Martinez‐Gomis, J. (2020). Reliability and validity of T‐scan and 3d intraoral scanning for measuring the occlusal contact area. *Journal of Prosthodontics*, *29*(1), 19-25).

- In addition, the authors can discuss the indications and limitations of each system in checking the occlusion. This comparison can be done using a table. The following articles can be helpful: Lee *et al*., 2018 ^3^ ; Cheng *et al*., 2018 ^4^ ; Solaberrieta *et al*., 2016 ^5^ .

**Author response: **The use of the T-Scan system provides data for more in-depth occlusal analysis. Through this study, it is possible to determine whether the occlusion is balanced and also the sequence, strength, and location of the occlusal contacts. 3Shape software does not have these features. Our opinion on the benefits of using the computerized analysis system is confirmed by other authors (Kerstein RB. Current applications of computerized occlusal analysis in dental medicine. Gen Dent. 2001; 49: 521-530; Buduru S, Mesaros A, Talmaceanu D., Baru O, Ghiurca R, Cosgarea R. Occlusion in the digital era: a report on 3 cases. Medicine and pharmacy reports. 2019; 92 (3): 78). Therefore, we recommend that, if technically possible, you should not rely solely on the information provided by the intraoral scanner, however accurate it may be.

# Peer review: Reviewer response for version 1

**DOI:** 10.5256/f1000research.76564.r95636

**Published:** 2021-10-21

**Authors:** Bozhidar Yordanov

**Affiliations:** 1 Department of Prosthetic Dental Medicine, Faculty of Dental Medicine, Medical University – Sofia, Sofia, Bulgaria

The currentness of the subject discussed in this paper is determined by the contemporary requirements for optimum rehabilitation of the masticatory system in patients with bruxism. Splint therapy of bruxism is still the basic method of treatment to relieve specific clinical symptoms - temporomandibular disorders, muscle hyperactivity, and lost esthetics as a result of abrasion of tooth occlusal surfaces. Contemporary digital methods for the examination of occlusion and fabrication of occlusal splints contribute to the precision and better final results following occlusal therapy. Patient examination, treatment planning, digital process for fabrication of the splint, adjustment of occlusal contacts using conventional and modern methods and technologies, discussion of the results and conclusions are sufficient enough for understanding diagnosis, planning, treatment of bruxism and to be useful for dental practitioners.

 Critical notes:

1. The background of the case’s history and progression is not completely described in sufficient detail.
2. The scientific value of this paper will be increased by replacing some specific terms with more accurate ones: occlusal splint instead of stabilization splint; dental medicine instead of dentistry; bilaterally balanced occlusion instead of balanced occlusion; adjustment of occlusal contacts instead of articulation; retail worker instead of shop assistant; opposing teeth instead of antagonists; anterior instead of frontal; occlusal interferences instead of blocks.
3. In the second section of the Discussion, probably there is a mistake: "Stabilization splints for patients with bruxism aim to RAISE TMJ pain, muscle hyperactivity...".  Splints are used to relieve pain, not to raise it.

The critical notes above do not decrease the scientific and practical value of the presented article.

Are enough details provided of any physical examination and diagnostic tests, treatment given and outcomes?

Yes

Is the case presented with sufficient detail to be useful for other practitioners?

Yes

Is sufficient discussion included of the importance of the findings and their relevance to future understanding of disease processes, diagnosis or treatment?

Yes

Is the background of the case’s history and progression described in sufficient detail?

Partly

Reviewer Expertise:

Prosthetic Dental Medicine and Dental Implantology

I confirm that I have read this submission and believe that I have an appropriate level of expertise to confirm that it is of an acceptable scientific standard.

# Response

**Published:** 2021-12-02

**Authors:** dobromira shopova

Dear Reviewer, 

 Thank you for your advices! I will correct them in the final version of the article!
